# Influences on Modern Multifactorial Falls Prevention Interventions and Fear of Falling in Non-Frail Older Adults: A Literature Review

**DOI:** 10.14740/jocmr1874w

**Published:** 2014-07-28

**Authors:** Ulla Svantesson, Buki Babagbemi, Lakicia Foster, Marie Alricsson

**Affiliations:** a Institute of Neuroscience and Physiology/Physiotherapy, The Sahlgrenska Academy, University of Gothenburg, SE 405 30 Goteborg, Sweden; b Mid Sweden University, Swedish Winter Sports Research Centre, Department of health Science, SE 83125 Ostersund, Sweden; c Johns Hopkins University School of Nursing, 525 North Wolfe Street, Baltimore, MD 21205, USA

**Keywords:** Self-efficacy, Multidisciplinary, Physiotherapy

## Abstract

This review explores underlying features that may influence fear of falling and the effectiveness of multifactorial falls prevention programs in community dwelling non-frail adults aged 65 and older. It also examines the interrelationship between fear of falling and multifactorial falls prevention interventions. A literature search of medical databases was conducted to identify articles that address the fear of falling and multifactorial programs as either a primary or secondary component of their findings. Multifactorial interventions were assessed in terms of their program content, design, demographics, implementation techniques, and cost-effectiveness. Falls are a common, but preventable, cause of morbidity and injury in older adults 65 and over. In addition to physiological variables, fear of falling and self-efficacy are psychosocial factors that impact the incidence of falls in this population. Addressing fear of falling in addition to physiological parameters may influence the success of multifactorial falls prevention programs for adults 65 and over.

## Introduction

Every year approximately one-third of community dwelling adults 65 and older experience a fall [1-5]. Falls account for the major cause of accidental injuries and injury-related deaths in this age group [3, 5, 6]. The risk of falling increases with aging and as co-morbid health conditions accumulate, about half of older persons over the age of 80 will fall annually [1, 7]. An occurrence of a fall usually results in a future fear of falling [8, 9]. However, the fear of falling may also be present in older adults who have never fallen [9]. Debilitating consequences may occur from the fear of falling [4, 9]. Studies show that the fear of falling may lead to a continual, complex decline in older adults that includes a “loss of confidence, restriction of physical activities and social participation, physical frailty, falls and loss of independence” [9]. Apart from these debilitating effects, the fear of falling may also have financial implications for healthcare systems and the general public [9].

A recent Cochrane systematic review comprising mainly of single and multifactorial intervention found that multifactorial programs that include an individual risk assessment were effective in reducing the rate of falls but not the actual risk of falling [4]. Many falls prevention studies in community living older persons have focused on the effectiveness of different interventions in addressing the problem of falls [3, 4, 7], but less data are available on the specific components that influence their effectiveness [10]. The purpose of this literature review is to examine what influences multifactorial falls prevention programs and fear of falling, and the interrelationship between fear of falling and the effectiveness of multifactorial falls prevention programs for older, non-frail, community dwelling adults aged 65 and older.

## The Literature Search and Inclusion Criteria

The following databases PubMed, CINHAL, Cochrane and Scopus were searched using the following terms: falls prevention, fear, fear of falling, multifactorial, interventions, multifactorial interventions, functional ability, aged adults, community dwelling older adults, and older adults. All PubMed, CINHAL, Cochrane, and Scopus searches were limited to peer-reviewed research articles with abstracts. Additionally, articles were suggested through electronic redirection to SciVerse from PubMed. These articles were then reviewed and assimilated into the literature review as appropriate. Only articles written in English and published within the last 5 years (2008 - 2013) were used to abstract results. Study population inclusion criteria were participants aged 65 and older who were community dwelling, living at home and receiving partial or occasional assistance or support, functionally independent, or possibly living with some co-morbid conditions such as osteoporosis and hypertension. Exclusion criteria included older adults under the age of 65 and those who were frail, institutionalized, cognitively impaired (such as having dementia), low functioning, totally dependent, or had neuromuscular disorders such as Parkinson’s disease. A target population of 65 and older was chosen for this review because there are many statistical references found regarding the incidence of falls in this age group [1-6].

## Literature Review and Discussion

### Description of studies

**Figure 1:**
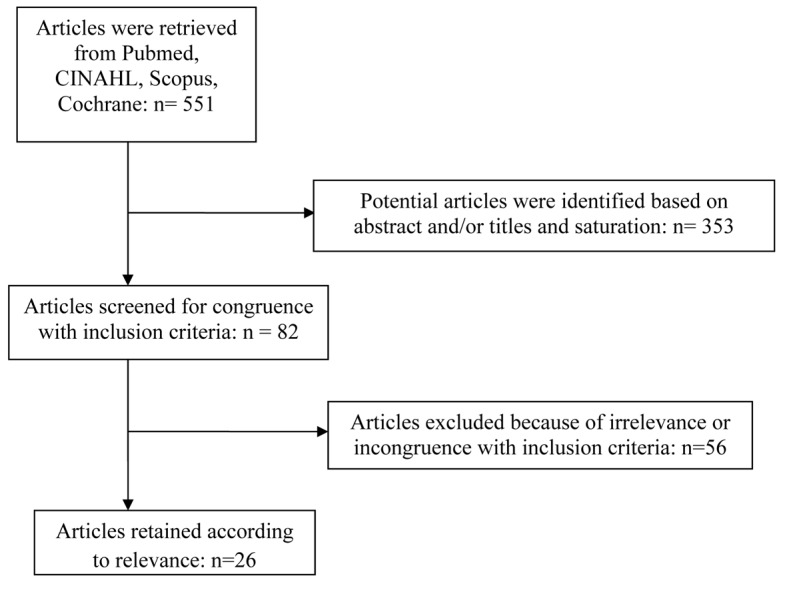
Article screening flow chart.

A total of 551 articles were retrieved from all databases searched for this review. Articles were selected for evaluation based on the relevance of their abstracts and titles to the research foci, fear of falling and multifactorial falls prevention programs targeted at community dwelling and functionally independent older adults over the age of 65 (Fig. 1). This study combined results from both quantitative and qualitative research studies to explore what influences multifactorial falls prevention programs and fear of falling and the interrelationship between fear of falling and the effectiveness of multifactorial falls prevention programs for older, non-frail, community dwelling adults aged 65 and older. A total of 26 articles were selected for the review. Twelve articles met results inclusion criteria. One addressed fear of falling through the falls efficacy scale (FES) [11], while three others addressed fear of falling related to balanced control [12-14]. Two focused on fear of falling in relationship to multicomponent falls prevention intervention programs [10, 15]. Three focused on the effectiveness of multifactorial interventions in comparison to other interventions such as physical exercise, guidance counseling, individual focused advice and standard care [3, 5, 7]. Two articles addressed multifactorial programs from the standpoint of cost-effectiveness [16, 17]. One study explored how older adults cope with falls and what motivates them to participate in falls prevention programs [18]. Analysis of the results revealed interrelationships between definitions, concepts, program constructs, designs, strengths, weaknesses, self-efficacy, and complexity of multifaceted dimensions related to fear of falling and multifactorial falls prevention programs for non-frail, functionally independent, older adults aged 65 and over (Fig. 1).

### Self-efficacy, fear of falling and falls

Self-efficacy may be defined as one’s belief in his or her ability to accomplish a certain task or behavior, or to do so in a specific situation [19]. Literature further describes that theoretically one’s self-efficacy is informed by one’s performance accomplishments, verbal encouragement, vicarious learning, and one’s affective state [19]. One’s belief in their ability to complete a given task is influenced by their past accomplishments, what they have seen others accomplish, verbal encouragement they have received, and their psychological disposition as they relate to the task [19].

Fear of falling is explained as poor self-efficacy related to falls avoidance during non- harmful and everyday activities [19]. Among older community dwelling adults, fear of falling has a reported prevalence between 20% and 83%, and has been described as a major cause of morbidity leading to reduced physical activity, functional decline, and loss of independence [20]. In this way, fear of falling may lead to frailty, social isolation, and diminished quality of life for older adults [19]. On the other hand, improved self-efficacy may lead to greater endurance, positive disposition, and energy during non-hazardous, ability appropriate activity [21].

Falls may be defined as unintentionally decreasing in spatial level or coming to rest on the ground independent of an intrinsic event or hazard [12, 15]. Research suggests that the relationship between falls and fear of falling is still not fully clear [22]. In an effort to further elucidate this relationship, versions of the FES have been used [11]. Studies have indicated that the falls efficacy scale international (FES-I) and its revised versions are reliable resources for measuring fear of falling and the potential for future falls [11]. Results of an evaluation of the Turkish version of the FES-I indicated that those with reported fear of falling also scored higher on the FES, indicating that the scale was valid for measuring fear of falling and identifying those at risk for future falls and functional decline [11]. Other studies have used multidimensional falls prevention programs to indicate, elucidate, and prevent the effects of fear of falling on actual falls incidence [14, 15, 18].

### Multifactorial falls prevention programs: conceptual underpinnings, program contents, and program designs

Some research indicates that falls are the result of multivariate, interrelated risk factors and situations [5, 23]. Associated risk factors may be intrinsic, such as demographic or biological, or extrinsic, such as behavioral or environmental [23, 24]. Due to this multivariate nature of falls, some research suggests that interdisciplinary and early prevention may be most successful at addressing and limiting the risk factors associated with falls [1, 23]. Among the dimensions that research suggests are pivotal to the success of fall prevention programs for non-frail, older adults 65 and over are recommendation of the program by their general physician, verbal support for the program from one’s relatives, and motivating the individual to believe that the program is advantageous and socially beneficial [18]. This is particularly important as falls are considered the leading cause of unintentional injury and injury-related death in persons 65 and over [3, 5, 6, 9]. Research also indicates that 30-40% of this population fall a minimum of once a year [1, 2, 4-6]. Expert opinion also suggests that for a multifactorial falls prevention program to successfully reduce the occurrence of falls, it must successfully address three constructs: content, process and choice of target group [25]. In this context, content refers to the components of the program; process refers to program delivery and means of delivery; and choice of target group refers to the selection of a population that is appropriate to receive and fully participate in the program [25].

Studies promoting multifactorial programs vary in their recommended approach to falls prevention. One study focusing on multiple-fall-related domains examined the interrelationship between physical and psychological components (including strength training, endurance, balance, and falls risk education) of falls prevention in an effort to determine what components yield the most positive outcomes among non-frail older adults over 65 [15]. In regards to fear of falling, the experimental group receiving physical and psychological interventions did not show a statistically relevant improvement in psychological dimensions of exercise and falls prevention, such as balance self-efficacy [15]. As it relates to program content, this study explored specifically what components make the most effective falls prevention program for adults over 65, highlighting the importance of elucidating the ingredients of a successful intervention.

The conceptual premise behind another falls prevention program was that fear of falling contributes to the risk of falling, but does not equal an actual risk [14]. The program focused on the effects of fear on postural synergy and proposed that fear of falling is related to anxiety, symptoms of depression, diminished quality of life, and unnecessary activity avoidance in older adults [14]. While a causal relationship between fear of falling, gait, and falls could not be concluded in the study, the results of the study did indicate a direct correlation between fear of falling and prolonged anticipatory postural adjustment (APA) phase during gait initiation which is associated with deficient balance [14]. In this study, participants with fear of falling were found to have longer APA phases while completing dual tasks (counting backwards while awaiting a visual cue to initiate walking) than did dual task control participants who lacked the fear of falling component [14]. This program reflected an important emphasis on fear of falling as a conceptual underpinning in addition to physiological parameters when determining the content and design of a multifactorial falls prevention programs.

A qualitative study of adults 65 and older explored the impact of older adults’ impression of the significance of falls, personal coping mechanisms, and factors that motivate them to participate in falls prevention programs [18]. Participant motivation to engage in falls prevention programs included psychosocial parameters, such as participant perception of falls prevention program as enhancing their autonomy, including social and physical benefits, or being approved of by a spouse or general physician [18]. Importantly, the study pointed out that multifactorial falls prevention programs would benefit from taking into consideration the older person’s specific perception of fall significance and their personal priority to experience autonomy, social support and relatedness, and a sense of competence [18]. Falls prevention programs that promote these physical and psychosocial dimensions in the design of the program may experience improved participation and outcome. Personalized program design and content are another important aspect of effectively addressing fear of falling and multifactorial falls prevention programs in the targeted population.

In a study measuring the effects of interventions to decrease fear of falling in older adults, involving participants over 60 years of age, fear of falling was found to be significantly reduced in interventions that used a combination of exercise and education, those measured after 4 months, and those in home-based settings [26]. The latter results yielded the smallest effect size [26]. Overall, the results suggested that interventions that include physical and psychological components, sufficient time for initiation, and a familiar setting have the greatest impact on falls reduction [26]. Due to the age proximity of the study population, these results may be possibly applicable to falls prevention programs for older community dwelling adults over the age of 65, but further research would need to be conducted to determine the verity of this statement. Among other variables, time in particular may be a key component to carefully consider in program design. In a meta-analysis of the target population, in which exercise-alone and multifactorial interventions were examined, interventions that lasted less than 12 months, including one multifactorial program, were found to be more effective at reducing falls in community dwelling older adults [7].

### Multifactorial falls prevention programs: program demographics, implementation techniques, and cost-effectiveness

In the study above, multifactorial interventions targeting clients 80 years and older were generally less effective compared to programs for adults averaging age 70 [7]. This highlights the significance of considering population demographics when developing, implementing, and evaluating falls prevention programs for adults 65 and older.

In another study, a Dutch multicomponent cognitive-behavioral group intervention was adapted from a US model to address fear of falling, fall risk, and actual activity performance [10]. The Dutch intervention group demonstrated more positive, although not statistically significant, decreases in falls compared to the intervention group in the US study. This indicates the significance of population demographics and implementation techniques.

In the Dutch implementation, researchers reported through their data a more rigorous application of their program compared with the US intervention [10]. Further, the Dutch version showed longer lasting and more permeating effects on the intervention group with supplementary intervention effects in those who adhered to the program, while the US version of the intervention demonstrated durable intervention effects primarily in participants of the intervention group who adhered strictly to the program [10]. This relatively more successful outcome of the Dutch version, despite the similarities of the programs, may be attributable to their more rigorous approach as well as to the use of weekly (instead of biweekly) sessions and the addition of a booster session [10].

Finances are another dimensions that may impact multifactorial falls prevention programs [16, 17]. In a multifactorial falls prevention program for war veterans that included individualized risk assessment, the net benefit and incremental cost of the program was evaluated and compared to usual care costs and net benefit [17]. The results of the study demonstrated that there was no significant difference in falls related outcomes in the intervention group compared to the control group [17]. Further, costs for the intervention group were significantly higher, making the overall program financially inefficient and not cost-effective compared to usual care or other community-based primary care services [17].

In another cost-effectiveness analysis of falls prevention programs for Australians 65 and over, Tai Chi was found to be more effective and cost-effective than all other examined interventions, including multifactorial interventions programs [16]. A univariate sensitivity analysis also revealed that community dwelling falls prevention interventions that excluded fear of falling as an integral component of the program were less cost-effective overall than interventions that included fear of falling as a component [16]. In both of these studies, multifactorial falls prevention programs were found to be not cost-effective compared to other programs.

### Multifactorial falls prevention programs: evaluation of program effectiveness and innovations

The challenge of research is to uncover precisely which domains and components of intervention programs best meet the falls prevention needs of older adults who are at risk for falls [15]. In fact, there is a paucity of research that clearly articulates which multiple domains are most effective in falls prevention programs for older adults [10].

The Dutch intervention previously described sought to improve participant self-efficacy related to falls, one’s control over falling, risk perception regarding falls, and falls outcome expectations [10]. The intervention engaged four strategies followed by appropriate behavior modification to accomplish this goal: restructuring participants’ misconceptions so that they perceived falls as controllable, setting realistic goals for safely increasing activity, implementing home improvements that reduced falls, and using strength and balance activity promotion [10]. The researchers concluded that the multicomponent cognitive behavioral intervention largely influenced the durable, positive intervention outcomes on fear of falling, associated activity avoidance, and falls rate reduction in their community dwelling older adults [10]. Also, falls data that were systematically obtained for safety purposes showed a reduction in falls rates and suggested that further research may prove this approach as instrumental in reducing falls in future balance training programs [10]. These results demonstrate that inclusion of psychosocial components in multifactorial falls interventions may impact effectiveness. Specifically, cognitive-behavioral designs in falls prevention programs may also effectively influence falls reduction.

In a Spanish multifactorial falls prevention study, intervention participants received individual advice, information leaflets, a physical exercise workshop, and home visits [5]. Compared to the control group, which received brief intervention, the group who received the multifactorial intervention experienced statistically significant reduction in fear of falling and actual fall occurrence at home compared with the control group [5]. This is another example of a multifactorial intervention that was successful in addressing falls and fear of falling in community dwelling older adults. It is important to note, however, that this intervention was found to be no more successful at reducing overall falls risk than the control [5]. This raises the question as to why multifactorial interventions are sometimes not successful at reducing falls occurrence or fear of falling among community dwelling older adults.

Researchers have reported that exercise alone interventions are five times more effective in falls reduction for recurrent, community dwelling fallers than multifactorial interventions [7, 13]. Research also suggests that it is difficult to assess all multifactorial interventions to ascertain the root causes of negative results [25]. To best address the success or failure of a program, it is suggested that multifactorial falls intervention programs should be assessed in the context of the three aforementioned constructs of content, process, and choice of target group [25]. Further, to what extent each construct was applied to the intervention and the possible benefits of adjusting the degree of application of one construct in relation to the others may also prove vital in the assessment and improvement of the multifactorial intervention [25]. It is also important when comparing multifactorial and usual care falls prevention programs that evaluators explore the components of both programs because if both programs include multifactorial components, the comparative results may not yield high significance because of overlap in equally positive effects of the multifactorial components [25].

In a randomized control trial in Finland [3], aging participants who had fallen within the past year were involved in a multifactorial risk analysis intervention that included falls prevention guidance and counseling, geriatric assessment, home exercises, group lectures, and psychosocial groups. When compared to the control, the multifactorial intervention was found to be ineffective in reducing the incidence of falls in community dwelling older adults who had fallen at least one time in the previous year [3]. Limitations of the study were potentially opportunistic recruitment and that the study may have been underpowered [3]. Although the general intervention effect was smaller than the researchers anticipated, overall, in participants with a higher number of depressive symptoms, falls incidence was reduced, and depressive symptoms were found to decrease with increased frequency of exercising (which was linked to improved physical function and falls reduction in program participants) [3]. While results of targeted outcomes were not statistically significant, positive effects on various aspects of falls prevention in the program did occur, as was evidenced by the subgroup analyses. These findings highlight the specific nature of multifactorial intervention programs. They also elucidate the importance of developing multifactorial falls prevention programs for older adults that are relevant to the population, are sufficiently powered, are suitable for the targeted outcomes, and are appropriately implemented to yield statistically significant results.

In an innovative, original research program, a recently validated, reliable, virtual-reality system called the balance rehabilitation unit (BRU) community dwelling older adults completed 6 weeks of biweekly, 30-min training sessions comprised of visual-vestibular rehabilitation and postural training exercises [12]. At 9-month follow-up, compared to the usual care only group, BRU training group showed significantly lower falls incidence and lower levels of fear as measured by the SAFE scores, and participants also reported greater motivation and enjoyment of the program compared to usual care physical therapy [12]. Also, with the exception of software and equipment, the intervention reported costs similar to other falls prevention programs that include regular physical therapy sessions [12]. In this novel study, not only were the researchers successful in reducing participant fall incidence and fear of falling, they were also able to foster participant motivation to participate and remain in their multifactorial falls prevention program. The preliminary success of this study demonstrates that innovative, falls prevention programs can be cost-effective, address fear of falling, have positive intervention effects, and retain participant interest.

## Conclusions

Falls are a common cause of morbidity and injury in adults 65 and over. Research demonstrates that in addition to balance control and other physiological variables, self-efficacy and fear of falling are psychological dimensions that influence the incidence and frequency of falls in older adults. Fear of falling in itself is understood in the context of the psychosocial and behavioral experience of the individual. From this perspective the nature of falls is multifaceted, including psychosocial, behavioral, cognitive, and physiological components that are often as complex and unique in construction as the individual faller. Outcomes of falls prevention programs, then, may be most successful when they carefully take into consideration the physiological and psychosocial experience of the individual faller, including fear of falling and self-efficacy. While falls and fear of falling are very important areas of geriatric health, there appears to be a paucity of modern, relevant research that conclusively elucidates what factors make a cost-effective, successful, generalizable multifactorial falls prevention program that improves falls incidence and fear of falling among older adults 65 and over [12]. A major limitation of this study was restriction of the review population to 65. This yielded very narrow results that precluded the abstraction of overlapping data.
